# No evidence of *Borrelia mayonii* in an endemic area for Lyme borreliosis in France

**DOI:** 10.1186/s13071-017-2212-7

**Published:** 2017-06-05

**Authors:** Pierre H. Boyer, Sylvie J. De Martino, Yves Hansmann, Laurence Zilliox, Nathalie Boulanger, Benoît Jaulhac

**Affiliations:** 1 Early Bacterial Virulence: Lyme borreliosis Group, Université de Strasbourg, CHRU Strasbourg, Fédération de Médecine Translationnelle de Strasbourg, VBP EA 7290, F-67000 Strasbourg, France; 2 French National Reference Center for Borrelia, Hôpitaux Universitaires de Strasbourg, Strasbourg, France; 3 Department of Infectious and Tropical Diseases, Hôpitaux Universitaires de Strasbourg, Strasbourg, France

**Keywords:** Lyme borreliosis, *Borrelia mayonii*, *Borrelia burgdorferi* (*sensu lato*), Fever

## Abstract

**Background:**

*Borrelia mayonii* is currently the latest species belonging to the *Borrelia burgdorferi* (*sensu lato*) complex to be discovered. Interestingly it is involved in human pathology causing a high fever. We looked for its presence in post- tick bite febrile patients as well as in *Ixodes ricinus* ticks in an endemic area of France.

**Results:**

After ensuring that our molecular technics correctly detected *B. mayonii*, 575 patients and 3,122 *Ixodes ricinus* nymphs were tested. Neither *B. mayonii* nor another species of the *B. burgdorferi* (*s.l*.) complex previously not reported in Europe has been identified.

**Conclusions:**

For now, *B. mayonii* seems to be an epiphenomenon. However, its discovery broadens the etiology of post-*Ixodes* bite febrile syndromes.

## Background

Lyme borreliosis is reported as the most common tick-borne disease in the United States [1] and all the northern hemisphere. Since the early research of Baranton and colleagues [2], several species belonging to the *Borrelia burgdorferi* (*sensu lato*) (*s.l*.) complex have been described. Four are currently the main etiological agents of human Lyme borreliosis in Europe [1], and *Borrelia burgdorferi* (*sensu stricto*) (*s.s*.) was considered for a long time as the only species isolated from human clinical samples on the American continent [3, 4]. The recent discovery of *Borrelia mayonii* [4] questions this idea. This newly identified bacterium seems to display unusual clinical characteristics. Unlike the other species of the *B. burgdorferi* (*s.l*.) complex, uncommonly high fever due to unusual high spirochetemia as well as cutaneous rashes was observed in patients infected by *B. mayonii* [4]. It is, therefore, the first time that a member of the *B. burgdorferi* (*s.l*.) complex had been shown to induce high fever as reported for relapsing fever *Borrelia*, thus expanding the field of symptoms found in Lyme borreliosis. We, therefore, investigated its potential presence in patients presenting with fever after a suspicion of tick bite in Alsace, a French region endemic for Lyme borreliosis [5], and also in *Ixodes ricinus*, the vector of *B. burgdorferi* (*s.l*.) in western Europe [1].

## Methods

DNA extracts from whole blood samples of 575 febrile patients sent to our laboratory between January 2010 and July 2016 were studied. All patients included in this study hailed from northeastern France and developed either a fever above 38 °C within three weeks after a tick bite or fever of unknown origin with exposure to a tick bite in an endemic area of Lyme borreliosis during spring and summer (April to October). All the subjects enrolled in this study completed a written informed consent. In parallel, 3,122 *I. ricinus* questing nymphs collected monthly by flagging in four separate locations in Alsace from April 2013 to December 2015, were investigated.

Both patients and ticks were tested for *B. burgdorferi* (*s.l*.) using an in-house Taqman**®** real-time PCR assay [6], targeting a conserved region of the flagellin gene (*fla*) of the *B. burgdorferi* (*s.l*.) complex. *Borrelia turdii* whole DNA at a concentration of 1 pg/μl was used as a positive control. The genotyping of *B. burgdorferi* (*s.l*.) species was performed on positive samples with another real-time PCR assay using hybridization probes targeting species-specific regions of the *fla* gene [6]. To ascertain that the techniques correctly detect *B. mayonii*, we firstly aligned our primers and probes *in silico* with the 476 bp fragment of the corresponding *fla* gene (GenBank KR154295.1). Then, the *B. mayonii* type strain ATCC BAA 2743 was cultured in the BSK-H medium (Sigma-Aldrich, Saint Quentin Fallavier, France). *Borrelia mayonii* whole DNA was extracted with the MagNA Pure system (Roche Diagnostics, Meylan, France). After extraction, DNA concentration was measured by NanoDrop 1000 Fluorospectrometer (Thermo Fischer Scientific, Villebon sur Yvette, France). To evaluate the sensitivity of our technique, a dilution range was tested.

## Results

Analyses of results showed that *B. mayonii* was correctly detected by our *B. burgdorferi* (*s.l*.) PCR assay up to a concentration of 2 bacteria/μL; similar results were found with the main pathogenic species. Moreover, *B. mayonii* is never misidentified by our typing assay and amplicon sequencing would have confirmed *B. mayonii* identification. Only one out of the 575 blood samples tested from the post-tick bite febrile patients was positive for *B. burgdorferi* (*s.l*.). This sample was further identified as being *B. afzelii* by our typing assay and confirmed by sequencing. Interestingly, this patient had a co-infection with the tick-borne encephalitis virus (TBEv). He had, without previous vaccination, anti-TBEv IgG and IgM and thus met the ECDC Laboratory criteria for a confirmed TBE case [7]. On another serum tested one month later, the anti-TBEv IgM had disappeared. The involvement of *B. afzelii* in clinical manifestations of this patient is questionable since TBEv infection is known to induce fever and headache [8]. Among the 3,122 tested nymphs, 12.7% were infected with *B. burgdorferi* (*s.l*.), and only *Borrelia* species commonly found in this area were identified, such as *B. afzelii*, *B. garinii* and *B. burgdorferi* (*s.s*.). All ticks were negative for *B. mayonii*, and no other *Borrelia* species was identified.

## Discussion

The discovery of *B. mayonii* questions the paradigm of the absence of fever in Lyme disease. Considered as unusual [1, 9–11], high fever is not a cardinal symptom of Lyme disease, but three out of the six patients previously described by Pritt and colleagues [4] had fever above 38 °C with even two exceeding 39 °C. This new clinical picture broadens the symptom spectrum of Lyme disease, justifying the extension of investigation to an additional member of the *B. burgdorferi* (*s.l*.) complex in febrile patients in France. Neither *B. mayonii* nor another new species of the *B. burgdorferi* (*s.l*.) complex has been identified in our patient cohort. For now, *B. mayonii* should be considered an epiphenomenon [4]. The vector competence of *Ixodes scapularis* has been clearly demonstrated [12], but the competence of *I. ricinus* and *I. persulcatus* for this new *Borrelia* species, remains to be proven. Nevertheless, potential new members of the *B. burgdorferi* (*s.l*.) complex could be responsible for high fever and should be included in post-tick bite febrile syndrome active surveillance. This new pathogen monitoring should be initiated on European, Asian and American continents along with other etiological agents of post-tick bite fever.

## Abbreviations

*fla*: Flagellin
